# What Students Think They Feel Differs from What They Really Feel – Academic Self-Concept Moderates the Discrepancy between Students’ Trait and State Emotional Self-Reports

**DOI:** 10.1371/journal.pone.0092563

**Published:** 2014-03-19

**Authors:** Madeleine Bieg, Thomas Goetz, Anastasiya A. Lipnevich

**Affiliations:** 1 Department of Empirical Educational Research, University of Konstanz, Konstanz, Germany; 2 Thurgau University of Teacher Education, Kreuzlingen, Switzerland; 3 Division of Education, Queens College and the Graduate Center, The City University of New York, New York, New York, United States of America; University of Akron, United States of America

## Abstract

This study investigated whether there is a discrepancy pertaining to trait and state academic emotions and whether self-concept of ability moderates this discrepancy. A total of 225 secondary school students from two different countries enrolled in grades 8 and 11 (German sample; *n* = 94) and grade 9 (Swiss sample; *n* = 131) participated. Students’ trait academic emotions of enjoyment, pride, anger, and anxiety in mathematics were assessed with a self-report questionnaire, whereas to assess their state academic emotions experience-sampling method was employed. The results revealed that students’ scores on the trait assessment of emotions were generally higher than their scores on the state assessment. Further, as expected, students’ academic self-concept in the domain of mathematics was shown to partly explain the discrepancy between scores on trait and state emotions. Our results indicate that there is a belief-driven discrepancy between what students think they feel (trait assessment) and what they really feel (state assessment). Implications with regard to the assessment of self-reported emotions in future studies and practical implications for the school context are discussed.

## Introduction

*Much of what we call emotion is nothing more nor less than a certain kind–a biased, prejudiced, or strongly evaluative kind–of thought. But emotions and behaviors significantly influence and affect thinking, just as thinking significantly influences what we call emotions and behaviors.* ([1]; p. 71)

The emotions that a student experiences whenever learning in school is involved has become a growing area of research in education and psychology and a focus of attention for scholars, policy-makers, and the public. A number of special issues in leading journals have been dedicated to the study of academic emotions [2]–[6], and emotional and social skills have moved to the center of current standards movements and legislation [7]. Further, a number of large-scale international assessments have integrated emotions and related constructs into their programs (e.g., PISA [8]). The increased interest in the study of emotions is not surprising, as students’ affect has been shown to relate to a wide range of important process and outcome variables in the academic context and emotions are seen as important outcome variables themselves. Variables related to emotions include learning strategies (for example self-regulated learning: [9]), academic achievement [10], [11], lifelong learning [12], and domain and career choices [13]. Overall, beyond intelligence and domain-specific skills emotions have been consistently shown to be important predictors of learning and achievement [14] and are considered to be valued educational outcomes.

A number of disciplines in psychology investigates emotions and uses various definitions of this multifarious construct [15]. Researchers working within the field of educational psychology conceptualize emotions as comprising several components (e.g., [16], [17]). These include the affective component (the core “feeling”), the physiological component (heart rate, skin conductance, etc.), the cognitive component (thoughts related to the emotion), the expressive component (facial expression, gestures etc.), and the motivational component (for example, intention to continue an activity). When investigating students’ emotions, most of the studies rely on questionnaires to capture students’ academic emotions ‘in general’, or habitual emotions (trait). Recently, however, the focus of research has been shifting toward assessing students’ state emotions in real-life context via the experience-sampling method [3]. The advantage of real-life assessments is in their higher ecological validity [18] as study participants are asked during their daily routines and not outside the context in question. In line with this assumption, initial investigations consistently reveal a discrepancy with regard to mean-level differences between trait emotions, and emotions that are currently experienced, or state emotions [19]. These theoretical developments coupled with empirical findings call for further investigation of differences between trait and state assessment methods.

### Assessing Trait and State Emotions: The Accessibility Model of Emotional Self-report

One reason for the found discrepancy between trait and state assessments of emotions may be attributable to the fact that trait emotions seem to be more strongly influenced by semantic knowledge than state emotions are. Robinson and Clore [19] synthesize the findings with respect to the discrepancy between trait and state emotional assessment and propose an accessibility model of emotional self-report. The authors distinguish between trait and state emotional self-reports by classifying them according to the respective memory systems. Trait emotions are semantic, conceptual, and decontextualized, whereas state emotions are episodic, experiential, and contextual [19]. It is further suggested that state emotions are directly assessed and thus influenced by situational cues, whereas in trait assessments it is individuals’ beliefs and semantic knowledge (i.e., generalized knowledge about which emotions should be experienced in certain situations) that affect outcomes of the assessment. As a result, there is an expected discrepancy between trait and state emotional assessments with traits relating stronger to subjective beliefs.

A number of studies have examined mean-level differences between trait- and state-based assessments of mood or emotions, sometimes reporting inconsistent findings (i.e., trait ratings being higher and lower than state ratings; e.g., [20], [21]). However, the vast majority of investigations show higher intensities of trait as compared to state emotions [22]–[24]. This discrepancy between trait and state emotional assessments has been termed *intensity bias* [23] or *impact bias* [24].

The study of differences between trait and state self-reports has not been limited to the area of emotions. Additional empirical support for the discrepancy between trait and state self-reports comes from a variety of branches of psychology with studies investigating how semantic knowledge influences this discrepancy. For example, one of the earlier studies revealed that recalled and actual symptoms of women’s menstrual cycle significantly differed, with women overestimating the severity of symptoms upon recall, as compared to their real-time ratings [25]. The authors found that the more female participants believed in the influence of menstruation on well-being the more they overestimated their recalled symptoms as compared to their state-rated symptoms. Similarly, Porter et al. [26] investigated how assessment of trait coping strategies was biased according to gender stereotypes compared to momentary assessment of coping strategies. Another example comes from van den Brink and colleagues’ study that compared individuals’ recalled and diary ratings of the severity of headaches [27]. In it, study participants reported higher intensity and duration of their headaches in the retrospective assessment, as compared to their ratings captured by diaries (real-time, state assessments). The results of these studies are relatively consistent: Trait assessments appear to be more strongly influenced by subjective beliefs as compared to state assessments, with traits being rated higher than states. Further, some studies provide initial evidence that this discrepancy can be explained by subjective theories that people hold.

These empirical findings indicate that trait emotions do not appear to be a good indicator of actual state emotions. Trait emotions are assumed to be influenced by subjective beliefs and are generally overestimated, as compared to state assessments [19]. The reported tendency for the individuals to rate trait emotions higher makes scientists question trait assessments’ ecological validity. The review of literature on emotions in educational psychology, however, shows that there is a clear preponderance of studies that employ trait-based emotional assessments. Critical remarks about trait assessments considered, one may wonder why trait measures are still used at all to assess emotions. In addition to favorable economic considerations, with trait assessments being far less costly than state assessments, various studies demonstrate that traits are stronger predictors of future behavior and future choices [24], [28], [29]. In the educational context these future choices could represent domain and/or career choices [13], [30]. Thus, the aforementioned findings indicate that trait and state assessments may not be used interchangeably and should be selected depending on a research question that researchers are attempting to answer [31]. The current study will provide additional evidence and offer further insight into the discrepancy between trait and state emotional assessments.

### Academic Self-concept as a Possible Moderator of the Trait-state Discrepancy

Researchers have been trying to identify variables that may explain the discrepancy between ratings of trait and state emotions, and found subjective beliefs to be particularly relevant [19]. For academic emotions, it is assumed that students’ self-concept belief is an important moderating variable. The importance of self-concept can be inferred from Pekrun’s control-value theory of achievement emotions [32], which stipulates that the component of control, commonly represented by academic self-concept, is one of the most prominent antecedents of academic emotions. Academic self-concept represents an important control belief in the school context and is defined as memory structure and representation of the abilities and competencies a person has [33]. It has been shown to be positively associated with positive emotions and negatively with negative emotions. Due to its prominent role in academic emotions, self-concept belief should be particularly effective in explaining the discrepancy between trait and state emotional assessments in a way that this belief more strongly influences trait emotional assessments but does not bias state emotional assessments.

To our knowledge, there is only one study that investigated the role of self-concept in explaining the discrepancy between trait and state emotions [22]. This study examined gender differences in trait and state mathematics anxiety and showed that despite similar state mathematics anxiety ratings girls report higher trait mathematics anxiety ratings as compared to boys. The discrepancy between trait and state mathematics anxiety in girls was partly explained by girls’ lower self-concepts thus showing the significant role that self-concept plays in clarifying existing differences between the two approaches to assessment.

Several other studies investigated the influence of self-esteem, a construct that is closely related to self-concept, on emotional ratings. Robinson and Barrett [34] conducted three studies examining links between self-esteem and emotional judgments. The authors found that people with high self-esteem tended to more positively rate their trait emotional experiences. State emotional assessments, however, were found to be unrelated to self-esteem. Another study showed that self-esteem influences recall of emotional experiences in a way that high self-esteem more strongly biases positive emotional recalls [35]. In sum, in line with the accessibility model of emotional self-report [19] the results of these studies found that trait reports were more strongly influenced by semantic knowledge as compared to state self-reports. Further, self-concept and self-esteem were shown to be potential moderators of the discrepancy between the two approaches to emotional assessment. The current study will attempt to further extend our understanding of the role that self-concept plays in explaining these trait-state differences.

### Aims of the Present Study

The aim of this study was to compare students’ trait and state emotional self-reports with respect to a possible discrepancy between the two approaches. We also wanted to investigate whether academic self-concept impacts the magnitude of the discrepancy between self-reported trait and state emotions.

Based on the findings of prior empirical studies [22], [23], we expect to find a discrepancy between the rated intensity of trait academic emotions and state academic emotions. We expect trait emotions to be rated higher than state emotions (intensity bias; Hypothesis 1). Beyond our attempt to replicate previous findings of the intensity bias in the academic context, we intend to explain the discrepancy between trait and state emotional assessments with students’ academic self-concept. We expect self-concept to positively predict the discrepancy between trait and state emotions in positive emotions and negatively predict it in negative emotions (Hypothesis 2). That is, as control positively relates to positive emotions and negatively relates to negative emotions we expect students’ self-concept beliefs to influence trait emotional assessments in the same direction.

Our study hypotheses were investigated in two samples from two different countries. Four emotions were examined: Two positive, activating emotions of enjoyment and pride, and two negative, activating emotions of anger and anxiety. These were chosen based on their high importance and frequently occurrence in the school context [11]. We investigated our hypotheses in the context of mathematics because several studies found that academic emotions are organized in a domain-specific way [36], [37]. As mathematics is one major domain in the school context, e.g., because of its importance for a wide range of professions, we assume that this is a good starting point to investigate the hypotheses.

To summarize, we were interested in examining differences between students’ trait and state emotional assessments of enjoyment, pride, anger, and anxiety in mathematics. We expected trait emotions to be rated higher than their respective state emotions. Furthermore, we investigated whether self-concept of ability can explain this discrepancy between the two assessment methods.

## Methods

### Ethics Statement

The procedures of both studies were in compliance with the ethical standards (Ethical Principle of the WMA Declaration of Helsinki) and were deemed appropriate by the Institutional Review Board of the University of Konstanz. Participation in both studies was voluntary. Written informed consent was obtained from all participants in Germany and Switzerland. Furthermore, parents of study participants were informed about the nature of the study and its procedure, and the heads of schools as well as mathematics teachers in both samples approved the study protocol. Once the data were collected and entered, all identifiers that could link individual participants to their results were removed and destroyed. Hence, all the analyses were conducted on anonymous data.

### Sample

Two samples were included in the current study. The first sample consisted of *N* = 94 German students of grade 8 (54.8%, *M_age_* = 14.30 years, *SD* = 0.51; 24 males) and grade 11 (*M_age_* = 17.57, *SD* = 0.58; 19 males) of 39 different classes (two to three randomly chosen students per class) from the upper track of the state school system in Germany (Gymnasium). The second sample included *N* = 131 9^th^ -graders from German-speaking Switzerland enrolled in 41 classes (three to four randomly chosen students per class; 44.3% male, *M_age_* = 15.67 years, *SD* = 0.64).

Although Germany and Switzerland are neighboring countries, there are several differences in their school systems that stem from rather unique educational traditions. One major difference is that students in Switzerland are separated according to ability tracks at a later point in time (usually after six years as compared to four years in Germany). Another notable difference has to do with the class size, which is usually smaller in Switzerland.

### Procedure

Students’ trait emotions and self-concept in mathematics were assessed via paper-and-pencil questionnaire that was administered by trained experimenters. The same items were used in the German and the Swiss sample. The procedure for the assessment of students’ state emotions was highly similar in the German and the Swiss sample and started right after the trait assessment. State data were assessed by employing a computer-based experience-sampling method [38]. In the German sample two to three randomly chosen students from each classroom were provided with a personal digital assistant (PDA). In the Swiss sample three to four students per classroom were provided with a PDA. The participants were asked to activate PDAs at the beginning of every mathematics class for a period of two regular school weeks in order to ensure ecological validity of state assessments.

The PDA randomly signaled within 40 minutes from the start of a lesson, prompting students to answer questions about their momentary emotions during that specific class. Therefore, our research design combines event-based and random sampling [18]. Students who took fewer than two assessments were excluded from the analyses. In total, this procedure resulted in *N* = 415 state measures with a mean number of 4.41 state assessments per student in the German sample and *N* = 749 state measures with a mean number of 5.72 state assessments per student in the Swiss sample. As a reliability measure for our mean state data, we calculated the intraclass correlation coefficient (ICC(2); see [39]). The ICC(2) ranged from 0.68 to 0.75 with anxiety having the lowest and pride having the highest value (ICC(2) = 0.70 for enjoyment; ICC(2) = 0.76 for pride; ICC(2) = 0.68 for anxiety; ICC(2) = 0.70 for anger) suggesting acceptable reliability.

#### Assessment of trait emotions

In both samples single items were used to assess the four trait emotions of enjoyment, pride, anger, and anxiety: ‘How much [EMOTION] do you generally experience during mathematics classes?’ The response format consisted of a 5-point Likert scale ranging from 1 (*strongly disagree*) to 5 (*strongly agree*).

#### Assessment of state emotions

State emotions were assessed using single items for each of the four emotions [parallel wording to trait assessment adjusted for the lesson: ‘How much [EMOTION] are you experiencing during this class?’; see [22]. The decision to use single items was due to practical reasons (e.g., minimizing lesson disruptions) and to avoid unintentionally evoking or changing emotions by the emotional assessment itself [40]. By assessing emotions with single items we could not explicitly assess the whole range of components of emotions (i.e., cognitive, motivational, etc.). However, we assume that our single items are still able to represent the five components of emotions while maintaining satisfying reliability for the assessment of the whole construct [41], [42]. Responses ranged from *strongly disagree* to *strongly agree* (5-point Likert scale). In the Swiss sample students were asked to report emotions they are experiencing *‘right now’* as compared to *‘during this class.’* This was the only difference in the assessment between the two samples.

#### Assessment of self-concept

Similarly to trait emotions, students’ academic self-concept was assessed via paper-and-pencil questionnaire. Three items for academic self-concept were adapted from the Self-Description Questionnaire (SDQ) [43]. Sample item includes: ‘I have always done well in mathematics.’. The total score was calculated by taking an average of the three self-concept items.

### Statistical Analysis

The main focus of our analyses was on the discrepancy between trait and state emotions and how this discrepancy is moderated by self-concept. For that reason, we combined trait and state emotion measures for each emotion into one variable and separated them in our analyses by introducing a dummy called “Trait” with state measures being coded as 0 (reference group) and trait measures coded as 1. In our analyses, we did not report gender as a possible moderator of the trait-state discrepancy. Significant gender differences in trait mathematics anxiety but not in state mathematics anxiety were found (as reported in the study of Goetz et al. [22]). However, as gender differences in emotions were not a major concern in the present study, we decided not to include it in our analyses. As trait and state emotion measures are nested within students, and students are nested within classes, our data reflect a three-level structure with measurement points nested within students and students nested within classes. Thus, the analyses were conducted via multilevel statistics using HLM 6.08 (Hierarchical Linear Modeling [44]).

The advantages of the multilevel statistical approach, as compared to other analytical strategies that have been used to study differences between trait and state emotions (e.g., [35]; mean-level differences and moderator analysis) is that we can account for the nested data structure (multiple measurement points per person and persons nested within classes) and for different numbers of measurement points per person (one trait measure but different number of state measures per person). This results in more adequate standard errors in statistical testing. Furthermore, while using this intraindividual analysis (trait-state discrepancy within each student), we assure that we do not commit an ecological fallacy and draw conclusions on the wrong level of analysis [45].

#### Level 1 variable

In order to test Hypothesis 1 (discrepancy between trait and state emotional assessments; 0 =  *state*, 1 = *trait*), we introduced the Trait dummy into all of our hierarchical linear regression models. Due to the coding of this variable, the intercept evaluated as γ_000_ describes the mean state emotion (i.e. the value if all predictors are zero). The effect of the Trait dummy (γ_100_) in our models can be interpreted as an indicator of the discrepancy between state and trait emotions. Significant positive effects of the Trait dummy indicate significantly higher trait ratings as compared to state ratings.

#### Level 2 variable

We further examined whether the discrepancy between trait and state assessments can be predicted by students’ academic self-concept in mathematics (Hypothesis 2). Therefore, we added self-concept as a *z*-standardized variable into our multilevel analyses as a predictor of the slope of the Trait dummy (slope-as-outcome model), which results in a cross-level interaction between Level 1 and Level 2 (Trait × Self-concept interaction; γ_110_). This interaction term represents the effect of self-concept on the amount of difference between trait and state emotion scores. Positive effects indicate that higher self-concept values are associated with higher discrepancies between trait and state assessments, whereas negative effects for the self-concept variable indicate smaller discrepancies. For the sake of completeness, self-concept was also introduced into the model to predict the intercept (γ_010_). This ‘main effect’, which indicates the prediction of state emotions by self-concept, however, was not of importance in our hypotheses testing.

The mixed model regression equation for Model 1 (combined model), used for each of the four emotions, is as follows:



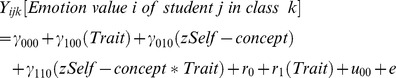



#### Level 3 variable

In addition to the Trait dummy and the self-concept variable, a dummy for either Switzerland (CH_Dummy; German model, Model 2) or Germany (DE_Dummy; Swiss model, Model 3) was introduced on the third level into the analyses to account for possible differences between the two samples. The difference between the two samples may be twofold. On the one hand, the samples were assessed in different countries (Germany vs. Switzerland) and on the other hand, slightly different instructions for state emotions assessment were used (‘in this class’ vs. ‘right now’). Thus, we present our analyses for the combined sample as well as for each of the two countries as a reference group (including a dummy variable for the other country, respectively). Coefficients for the interaction of each variable with the respective country dummy (i.e. Trait × CH_Dummy, γ_101_; Self-concept × CH_Dummy; γ_011_; Trait × Self-concept × CH_Dummy; γ_111_) indicate differences between the effect for the country as compared to the reference group, e.g. in the German model the dummy for Switzerland indicates differences between the effect for the Swiss sample compared to the German sample (reference group).

Hierarchical linear modeling, regression equations for Models 2 and 3.

Model 2– German model (German sample is reference group):



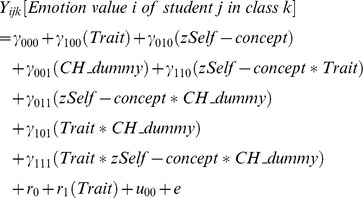



Model 3– Swiss model (Swiss sample is reference group):



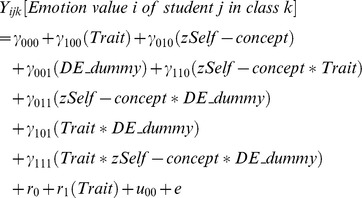



## Results

### Descriptive Statistics

The reliability of the self-concept scale was satisfying (German sample: α = .91; Swiss sample: α = .86). Table 1 shows means and standard deviations of variables for the combined sample and the German and Swiss samples separately. As expected, trait ratings are higher than state ratings for every emotion in both samples. The only exception is that state *enjoyment* in the Swiss sample was rated higher than trait *enjoyment*.

**Table 1:** Descriptive statistics.

	Combined sample (*N* = 225)				German sample (*n* = 94)				Swiss sample (*n* = 131)			
	Trait		State		Trait		State		Trait		State	
	*M*	*SD*	*M*	*SD*	*M*	*SD*	*M*	*SD*	*M*	*SD*	*M*	*SD*
Enjoyment	2.57	1.14	2.52	1.27	2.65	1.16	2.20	1.30	2.51	1.12	2.71	1.21
Pride	2.51	1.13	1.95	1.22	2.72	1.18	1.67	1.09	2.35	1.07	2.12	1.27
Anger	2.76	1.28	1.97	1.26	2.91	1.21	1.84	1.24	2.64	1.32	2.04	1.27
Anxiety	1.79	1.12	1.51	1.03	2.00	1.25	1.52	1.06	1.63	0.99	1.51	1.00
Self-concept	3.03	1.15	–	–	2.96	1.21	–	–	3.09	1.10	–	–

### Hierarchical Linear Regression

The results of the hierarchical linear regression for the four emotions of *enjoyment*, *pride*, *anger*, and *anxiety* are shown in Table 2. Further, the variance components are depicted in this table. We also calculated the explanatory power of self-concept with regard to the slope variance, that is, as a predictor of the trait-state discrepancy [46].

**Table 2:** Predicting emotions: results from multilevel modeling.

	Enjoyment			Pride			Anger			Anxiety		
	Model 1	Model 2	Model 3	Model 1	Model 2	Model 3	Model 1	Model 2	Model 3	Model 1	Model 2	Model 3
**Level 1**												
Intercept (γ_000_)	2.52***	2.25***	2.71***	1.95***	1.74***	2.10***	1.97***	1.79***	2.10***	1.50***	1.49***	1.51***
Trait (γ_100_)	0.05	0.42**	−0.22*	0.56***	1.02***	0.22*	0.77***	1.09***	0.54***	0.28**	0.48***	0.12
**Level 2**												
Self-concept (γ_010_)	0.19***	0.17**	0.17*	0.17**	0.19*	0.12	−0.14**	−0.22**	−0.10	−0.11*	−0.09	−0.13
**Cross-level interactions L1–L2**												
Self-concept × Trait (γ_110_)	0.23*	0.13	0.35**	0.30***	0.28**	0.36**	−0.34***	−0.32**	−0.33**	−0.33***	−0.41***	−0.24*
**Level 3**												
CH_dummy (γ_001_)		0.46***			0.37**			0.31*			0.02	
DE_dummy (γ_001_)			−0.46***			−0.37**			−0.31*			−0.02
**Cross-level interactions L1–L3**												
Trait × CH_dummy (γ_101_)		−0.63***			−0.80***			−0.55**			−0.36*	
Trait × DE_dummy (γ_101_)			0.63***			0.80***			0.55**			0.36*
**Cross-level interactions L2–L3**												
Self-concept × CH_dummy (γ_011_)		−0.01			−0.07			0.12			−0.04	
Self-concept × DE_dummy (γ_011_)			0.01			0.07			−0.12			0.04
**Cross-level interactions L1–L2–L3**												
Trait × Self-concept × CH_dummy/(γ_111_)		0.22			0.08			−0.01			0.17	
Trait × Self-concept ×/DE_dummy (γ_111_)			−0.22			−0.08			0.01			−0.17
**Variance components**												
Within-student (L1) variance ( 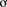 ^2^)	1.103	1.096		0.924	0.916		1.120	1.118		0.735	0.735	
Intercept (L2) variance (τ_00_)	0.419	0.391		0.460	0.429		0.419	0.404		0.254	0.255	
Slope (L2) variance (τ_11_)	0.174	0.122		0.252	0.128		0.243	0.173		0.258	0.217	
Intercept-slope (L2) covariance (τ_01_)	−0.269	−0.215		−0.320	−0.234		−0.230	−0.196		−0.109	−0.104	
Intercept (L3) variance	0.034	0.014		0.059	0.051		0.046	0.042		0.021	0.020	
**Explanatory power**	0.022	0.090		0.261	0.369		0.314	0.357		0.295	0.332	

Model 1 = combined model; Model 2 = combined dataset with German sample as reference group; Model 3 = combined dataset with Swiss sample as reference group.

Description of variables: Trait = Trait dummy (0 = state, 1 = trait); CH_dummy = Swiss dummy (0 = German sample, 1 = Swiss sample); DE_dummy = German dummy (0 = Swiss sample, 1 = German sample).

German sample: N_Level 1_ = 509; N_Level 2_ = 94; N_Level 3_ = 39; Swiss sample: N_Level 1_ = 880; N_Level 2_ = 131; N_Level 3_ = 41.

Explanatory power refers to the proportion of slope variance explained by the Level 2 and Level 3 predictors. The slope variance of the models in which no cross-level interaction is included was: τ_11_ = 0.178 for enjoyment in the combined model and τ_11_ = 0.134 in the German/Swiss model; τ_11_ = 0.341 for pride in the combined model and τ_11_ = 0.203 in the German/Swiss model; τ_11_ = 0.354 for anger in the combined model and τ_11_ = 0.269 in the German/Swiss model; τ_11_ = 0.366 for anxiety in the combined model and τ_11_ = 0.325 in the German/Swiss model.

*p<.05,

**p<.01,

***p<.001.

#### Model 1 – Combined model

In Model 1 the coefficient for the intercept (γ_000_) is to be interpreted as the mean emotion score when all other variables in the model are equal to zero. Thus, this represents the respective mean state emotion for a student who has a mean self-concept. The mean state score for the emotion of *enjoyment* was 2.52, 1.95 for *pride*, 1.97 for *anger*, and 1.50 for *anxiety*. The coefficient for the Trait dummy (γ_100_) is positive and significant for every emotion with the exception of *enjoyment*, for which no significant difference was found. Thus, with one exception, trait emotions are rated higher than state emotions (Hypothesis 1).

Regression weights for the Self-concept x Trait (γ_110_) interaction indicate the influence of self-concept on the discrepancy between trait and state emotional assessments. As expected, for *enjoyment* (.23) and *pride* (.30) the coefficients were positive, whereas for *anger* (−.34) and *anxiety* (−.33) the coefficients were negative. This suggests that high self-concept in mathematics is associated with higher discrepancies between trait and state *enjoyment* and *pride* and smaller discrepancies between trait and state *anger* and *anxiety* (Hypothesis 2).

#### Models 2 and 3 – German model and Swiss model

In order to account for differences between German and Swiss samples, we calculated models for each of the four emotions with a country dummy for Switzerland (CH_dummy; Model 2) and Germany (DE_dummy; Model 3). With regard to our first hypothesis, trait emotions were rated significantly higher than state emotions. In the German model (Model 2), coefficients for the Trait dummy (γ_100_) were 0.42 for *enjoyment*, 1.02 for *pride*, 1.09 for *anger*, and 0.48 for *anxiety*. Coefficients in the Swiss model were −0.22 for *enjoyment*, 0.22 for *pride*, 0.54 for *anger*, and 0.12 (*n.s.*) for *anxiety*. Hence, trait ratings were once again higher with the exception of *enjoyment* in the Swiss sample. Here, unexpectedly, the mean trait *enjoyment* was lower than the mean state *enjoyment*. Further, the discrepancy between trait and state *anxiety* was not significant in the Swiss sample. For each emotion, the discrepancy between trait and state ratings was found to be significantly lower in the Swiss sample (negative coefficient for Trait × CH_Dummy, γ_101_).

With regard to self-concept as a moderator of the discrepancy between trait and state assessments, the coefficients for the Trait × Self-concept interaction (γ_110_) were 0.13 for *enjoyment* (*n.s.*), 0.28 for *pride*, −0.32 for *anger*, and −0.41 for *anxiety* in the German sample (Model 2). In the Swiss sample (Model 3), the coefficients for the Trait × Self-concept interaction (γ_110_) were 0.35 for *enjoyment*, 0.36 for *pride*, −0.33 for *anger*, and −0.24 for *anxiety*. The strength of the moderation effect of self-concept on the trait-state discrepancy did not differ significantly between the two countries (all coefficients for Trait × Self-concept × Country dummy (γ_111_) were non-significant).

## Discussion

The aim of the present study was to investigate whether there is a discrepancy between trait and state academic emotions, and whether this discrepancy could be explained by students’ academic self-concept. The results of our study revealed a significant discrepancy between trait and state emotions in mathematics in a way that trait emotions were generally rated higher than state emotions with the exception of enjoyment and anxiety in the Swiss sample. Thus, our hypothesis about the discrepancy between trait and state mathematics emotions was generally supported (Hypothesis 1). This finding appears to be consistent with previous studies that have demonstrated an intensity bias in the prediction, recall, and evaluation of emotions in general [23], [29]. Due to the fact that we used parallel item formulations for trait and state emotional assessments, directly comparing mean-level differences was justified in our study. Despite the fact that both methods (i.e., trait and state) are routinely employed to assess students’ emotions, they obviously index different aspects of this construct. Thus, researchers and practitioners alike should refrain from drawing conclusions from mean-levels in trait assessments to mean-levels in state assessments and the other way around.

As predicted, self-concept moderated the magnitude of the discrepancy between trait and state emotional assessments (Hypothesis 2) with the exception of enjoyment in the German sample. Specifically, students with lower self-concept tended to more strongly overestimate their negative trait emotions (anger and anxiety) as compared to their actual state emotions. Conversely, students with higher self-concept tended to more strongly overestimate their positive trait emotions (enjoyment and pride) as compared to their actual state emotions in mathematics. Overall, trait emotional assessments seem to be influenced by subjective beliefs, and academic self-concept represents one of the most important beliefs in school. Our finding that self-concept moderates the magnitude of the difference between trait and state emotions is consistent with the view that trait emotions are more strongly biased by subjective beliefs and therefore capture *beliefs* about emotions and not necessarily individuals’ immediate, or state, emotions [19].

We just argued that it is not possible to draw conclusions from mean trait emotions to mean state emotions. However, knowing students’ academic self-concept should allow us to make a rough estimate of the similarity of trait and state emotional assessments and therefore the possibility to predict mean trait emotions from mean state emotions and vice versa. As trait emotions can be easily gauged, an estimate of the extent to which trait emotions reflect actual mean state emotions can be helpful, especially when more costly state assessments are not available. When talking about positive trait emotions, students with lower self-concepts seem to have a more ‘realistic’ estimate of their trait emotions, when state emotions are viewed as a benchmark for the ‘actual’ or ‘real’ emotions. The other way around, students with higher self-concepts seem to less strongly overestimate their negative trait emotions. Furthermore, it might be possible to find the self-concept threshold where the intensity of the respective trait and state emotion is estimated equally.

The explanatory power of self-concept in the prediction of the discrepancy between trait and state emotional assessments was.02 for enjoyment and.26 for pride, .31 for anger, and.30 for anxiety in the combined model. Overall, self-concept explained a substantial amount of variance in the discrepancy between trait and state assessments; however, it is only one of the beliefs which is important with regard to academic emotions. According to Pekrun’s control-value theory [32], value is another important appraisal antecedent that relates to the subsequent emotions. Intrinsic value reflects the value of an activity independent of the results. The lower explanatory power of self-concept in the trait-state discrepancy for the emotion of enjoyment may be attributable to the fact that enjoyment is one emotion, for which value appraisal may be more important than self-concept appraisal and thus, intrinsic value beliefs may be more predictive of the discrepancy between trait and state.

Related to this idea is a possible explanation of the finding that in the German sample, surprisingly, self-concept was not found to significantly moderate the magnitude of the trait-state discrepancy with enjoyment, even though the analyses comparing the two samples from Germany and Switzerland showed that self-concept was indeed a moderator with the other emotions studied. Thus, one reason for this unexpected finding could be the aforementioned importance of intrinsic value beliefs with regard to enjoyment. It is possible that value beliefs contribute much more to the trait-state discrepancy for enjoyment than does self-concept.

Another difference between the two samples was that in the Swiss sample average ratings of state enjoyment were higher than average ratings of trait enjoyment. In general, the discrepancy between trait and state emotional assessments was in all cases stronger in the German sample. The reason for this difference may be manifold. It is possible that cultural differences may lead to the difference. Another explanation may come from the different state item wording as enjoyment is a rather situation-specific emotion. Thus, the wording ‘How do you feel *right now*’ may lead to a stronger focus on the situation as compared to the specific math lesson. Future studies should employ identical items to compare results across samples and may use anchoring vignettes [47] when assessing differences in emotion self-reports across different countries.

To summarize, despite several unexpected results, our study revealed quite consistent findings with trait emotions being rated higher than state emotions and self-concept being a moderator of the trait-state discrepancy.

### Limitations and Future Directions

Our sample is limited to the upper track of the school system and only includes students from grade levels 8, 9, and 11. Future research may downward or upward extend our study and explore whether our findings generalize to students of different ages. Further, we only investigated our hypotheses in one specific domain, namely the domain of mathematics. This is justified given that academic emotions were found to be domain-specific with regard to mean-level differences [36]. Future research could test whether the findings of the present study generalize to other academic disciplines, which we assume should be the case as similar results were found in different contexts before [19].

Additionally, we only investigated the trait-state discrepancy with the emotions of enjoyment, pride, anger, and anxiety. Future research could include other emotions that are of high importance in the learning and achievement context. For example, boredom and relief are other relevant and frequently occurring emotions in school [48], [49]. Furthermore, it might also be relevant to investigate whether there are stable differences in the trait-state discrepancy for specific emotions. For example, in our study the trait-state difference was stronger for some emotions than for others. Examining possible reasons for these differences may help to further understand how trait-state discrepancy emerges.

We also used two different wordings for the state items in the two samples. Future studies should pay attention to the different formulation of items and investigate how this perhaps results in different outcomes, as manifested in larger or smaller discrepancies between trait and state emotional assessments.

Finally, our study investigated self-concept as a moderator of the trait-state discrepancy. As self-concept was shown to predict a significant amount of variance in the discrepancy between trait and state emotional assessments, it seems that self-concept is one of the most important variables with a high explanatory power. However, future studies may examine other possible moderators, such as value (e.g., intrinsic value for enjoyment) or stereotypic beliefs about emotions. It is possible that the effect of different moderators on the trait-state discrepancy may vary depending on the emotion being studied. For example, value beliefs could be more important in one emotion (e.g., enjoyment) than in another emotion (e.g., pride). Hence, investigating different combinations of discrete emotions and variables that may serve as moderators of trait-state discrepancy may prove to be a fruitful avenue for research. Additionally, future studies may also test for self-concept as a mediator of the relationship between emotional traits and states. According to the control-value theory, control appraisals, such as self-concept, are related to emotions, and feedback loops between emotions and appraisals exist. Thus, it would be useful to further investigate how state emotions contribute to the formation of one’s academic self-concept and how academic self-concept, in turn, influences one’s evaluation of trait emotions. That is, it would be helpful to examine the role of self-concept as a mediator of the relationship between emotional states and traits.

The results of the present study raise questions about the ecological validity of trait assessments as they seem to be strongly related to subjective beliefs and memory biases. In other words, they are different from state emotions, which are more immediately assessed in classroom situations. We would like to encourage researchers to differentiate between the two assessment methods and bear in mind that they cannot be used interchangeably. Hence, we implore investigators to choose one approach versus the other depending on a research question. As a possible limitation we would like to point out that state emotions assessed in our study are still self-reported emotions and not ‘actual’ emotions as it may be defined by neuropsychological or biopsychological perspective [50].

### Implications for Educational Practice

Explicating our findings from a practical perspective is particularly important: Students’ emotional beliefs seem to have strong impact on their future career choices more than their actual emotions. As traits affect future behavior [29] and domain and career choices in the school context [13], it is important to keep in mind that subjective beliefs may influence these choices, too. This may prevent students from proceeding careers in the respective domain.

Thus, when one is interested in far-reaching consequences of emotional beliefs, trait emotions are the assessment method one should use. In this way possible interventions can be derived. Students could be made aware of the possible discrepancy between their actual emotions and what they think about their emotions and how their beliefs may influence their career choices. Encouraging them to check whether their beliefs are consistent with their actual emotions can be a promising way to help students to go into mathematics careers [22]. In order to change subjective beliefs, cognitive interventions such as attributional retraining seem promising [51]–[53]. By prompting students to closely monitor their emotions we may help them to realize that they are not as anxious or angry as they believe they are.

With regard to this, teachers play a key role and they could be informed of the important influence of student self-concept on trait emotions and therefore its possible effects on individuals’ domain and career choices. From an intervention perspective, there are multiple programs aimed at fostering students’ self-concept [54]. It could be expected that a change in self-concept beliefs comes along with changes in emotional beliefs and may therefore contribute to basing future decisions on more realistic estimates of how one feels.

## Conclusion

The results of our study show that although trait and state assessments are intended to gauge the same construct, they are different. According to Robinson and Clore [19], state emotions more strongly refer to actual emotions (episodic, experiential, and contextual) whereas trait emotions refer to *beliefs* about emotions (semantic, conceptual, and decontextualized). As Ellis [1] noted in the initial quotation “[…] thinking significantly influences what we call emotions […]” (p. 71) seems to hold true at least for trait emotional assessments. This leads to the recommendation that researchers should clearly differentiate between the two assessment methods and assess emotions according to the main research question. Further, we found that the discrepancy between trait and state emotions is in part explained by students’ self-concept beliefs, with higher self-concept being associated with a stronger discrepancy of positive emotions and lower self-concept beliefs being associated with overestimation of negative emotions each compared to actual state emotions. In sum, it appears that what students think they feel (trait assessment) is not necessarily what they really feel (state assessment).
